# Early results of integrated malaria control and implications for the management of fever in under-five children at a peripheral health facility: a case study of Chongwe rural health centre in Zambia

**DOI:** 10.1186/1475-2875-8-49

**Published:** 2009-03-17

**Authors:** Pascalina Chanda, Busiku Hamainza, Susan Mulenga, Victor Chalwe, Charles Msiska, Elizabeth Chizema-Kawesha

**Affiliations:** 1 Ministry of Health Headquarters, Lusaka, Zambia; 2 Chongwe District Health Office, Ministry of Health, Lusaka, Zambia; 3 Tropical Diseases Research Centre, Ndola, Zambia

## Abstract

**Background:**

Zambia has taken lead in implementing integrated malaria control so as to attain the National Health Strategic Plan goal of "reducing malaria incidence by 75% and under-five mortality due to malaria by 20% by the year 2010". The strategic interventions include the use of long-lasting insecticide-treated nets and indoor residual spraying, the use of artemisinin-based combination therapies (ACT) for the treatment of uncomplicated malaria, improving diagnostic capacity (both microscopy and rapid diagnostic tests), use of intermittent presumptive treatment for pregnant women, research, monitoring and evaluation, and behaviour change communication. Financial barriers to access have been removed by providing free malaria prevention and treatment services.

**Methods:**

Data involving all under-five children reporting at the health facility in the first quarter of 2008 was evaluated prospectively. Malaria morbidity, causes of non-malaria fever, prescription patterns treatment patterns and referral cases were evaluated

**Results:**

Malaria infection was found only in 0.7% (10/1378), 1.8% (251378) received anti-malarial treatment, no severe malaria cases and deaths occurred among the under-five children with fever during the three months of the study in the high malaria transmission season. 42.5% (586/1378) of the cases were acute respiratory infections (non-pneumonia), while 5.7% (79/1378) were pneumonia. Amoxicillin was the most prescribed antibiotic followed by septrin.

**Conclusion:**

Malaria related OPD visits have reduced at Chongwe rural health facility. The reduction in health facility malaria cases has led to an increase in diagnoses of respiratory infections. These findings have implications for the management of non-malaria fevers in children under the age of five years.

## Background

Malaria accounts for 50% of outpatient attendances and 20% admissions in Africa [1]. In sub-Saharan Africa alone, annual malaria deaths are estimated at 1.3 million for all age groups. This has resulted into high expenditures on malaria control. WHO estimates show that countries may spend more than 40% of their health budget on malaria treatment and prevention [2]. This increase in health care expenditure and the negative effects of malaria on productivity slows down economic growth for these countries [3]. Not only does malaria have a negative impact on economic growth at national level [4], but it also affects the income levels of poor households [5]. Thus, integrated malaria control is high on the health agenda, as described in the Abuja declaration of 2000 [6].

In Zambia, malaria accounts for about 4 million cases (confirmed and unconfirmed) with approximately 6,400 deaths reported at health facilities country-wide [7]. The case fatality rates among hospital admissions are estimated to be 40/1,000 [8]. It is for this reason that malaria has taken centre stage in the National Health Strategic Plan (2006) and the fifth National Development Plan (2006). The main goal for malaria control is "*to reduce malaria incidence by 75% and under-five mortality due to malaria by 20% by the year 2010" *[9].

In order to achieve these goals, the Ministry of Health through the national malaria control programme is implementing integrated malaria control. The strategic interventions include the use of long-lasting insecticide-treated nets and indoor residual spraying, the use of artemisinin-based combination therapies (ACT) for the treatment of uncomplicated malaria, improving diagnostic capacity (both microscopy and rapid diagnostic tests), encouraging the use of intermittent presumptive treatment for pregnant women, research, monitoring and evaluation, and behaviour change communication. Financial barriers to access have been addressed, all malaria services (treatment and prevention) are offered free-of-charge. In April 2006, the government removed user fees in all 56 rural districts of Zambia [10].

All these interventions are known to be effective tools for malaria control. Further, studies in South Africa showed that ACT has the potential to reduce expenditure on malaria treatment [11]. Studies conducted elsewhere have demonstrated that both IRS and ITNs are effective prevention strategies in various settings [12-14]. In country studies have shown that ACT is a cost-effective treatment for malaria [15,16]. Utzinger and colleagues demonstrated that integrated malaria control resulted into better health for the community and higher productivity for the mining sector on the copperbelt in Zambia [17].

The Malaria indicator survey for 2008 report indicates that 75% Zambians own at least one ITN, 62% women received two or more doses of IPT, more than 77% of households in IRS designated districts were sprayed [18,19]. Further, in a health facility survey conducted in 104 health facilities in Zambia, it was found that 60% of the health facilities used ACT (artemether-lumefantrine), 73% had diagnostic capacity and at least 42% of children with uncomplicated malaria were treated with ACT [20]. An assessment on the impact of user fees removal on health facility utilisation found that from 2006 to 2007, there was an increase in utilisation of rural health facilities in the order of 7% and 55% for under-five and over-five years populations respectively [10]. Thus, both the population and health facility surveys have shown that malaria control interventions are reaching the service delivery points and the community members are having access. The malaria control programme has received financial and technical support from various multilateral, bilateral and private organisations.

In as much as evidence on process and outcome indicators exists, there has been less documentation of the impact of scaling-up malaria control in Zambia. As a result, a prospective study on the impact of scaling up malaria control is being conducted in various sites. This paper presents a case study of one particular rural district in Zambia where malaria control has began to show positive results at health facility level.

## Methods

### Study sites

The study was conducted in Chongwe district at Chongwe rural referral health centre. Chongwe district is located 35 Km from Lusaka city. This rural district has a population of 170,943 inhabitants. Most of the inhabitants are subsistence farmers. The district was among the first seven districts to pioneer use of ACT in 2003. In 2006, 31,322 people from 6,417 households were protected by IRS, while in 2007 the number of people protected with IRS was 63,307 (15,398 households). The total number of ITNs distributed in 2007 was 26,603. Chongwe rural health centre is a sentinel site for malaria information system and drug efficacy monitoring. The catchment area for Chongwe has also been selected as a site for demographic site surveillance.

### Study design and population

The study is part of a larger prospective evaluation of the impact of scaling up malaria control on health facility disease morbidity and mortality. All children aged five years and below who reported to Chongwe rural health facility with fever (or history of fever) from December 2007 to February 2008 were included in the study. All the children were screened by either a nurse or clinical officer on duty. Confirmation of malaria infection was performed by either RDT or microscopy, when RDT was used, a microscopy slide was taken for validation. Treatment of malaria patients was based on the treatment guidelines for malaria in Zambia [21].

The health workers involved in patient management included a nurse, a clinical officer, a laboratory technologist, dispenser and information officer. A community health worker was always available to assist with patient tracking and informed consent. The site was supervised by a team of two scientific officers and two medical doctors from Central and district level.

Non-malaria fevers were further investigated based on standard treatment guidelines for Zambia. All the drugs were supplied through routine mechanisms (Central Medical Stores) based on the essential medicines list for the country.

### Study procedures

The health workers at the health facility received an orientation prior on the commencement of the study on malaria treatment guidelines. Emphasis was placed on management of fever in children and more so the importance of management of non-malaria fevers. The district clinical care specialist and the health information officer were part of the team trained for the study. This was to enable them provide onsite supervision for both patient management and data management. No other interventions were applied.

All under-five children reporting to the out-patient department (OPD) of the health centre were identified and referred to the study screening room. A clinical officer or nurse then assessed the patient and performed a RDT test. Further management of the patient was based on the test result of the RDT. A microscopy slide was then taken for comparison reading by a laboratory technician on site. A sample of the slides was then taken to central level for quality assurance.

The patients were followed up on days 3, 7 and 14. The mothers were then requested to return to the health facility should the child develop any illness after that. Community health workers continued follow-up in the community to ensure that the children were not kept at home in case they fell sick.

### Treatment

The health facility receives a health centre drug kit from central medical stores. The drug kit contains all the drugs and supplies required for health service delivery for a second level health centre. All drugs are provided free to the patients. This is because the basic health care package in Zambia is free and, further, all rural health centres do not charge user fees as per policy recommendation [10]. The health worker decision on what medicine to prescribe to the children was largely based on their training and the stipulations in the standard treatment guidelines and depending on drug availability.

A medical doctor provided supervision on clinical aspects of patient management, a biomedical scientist supervised diagnostic procedures and the scientific officers provided oversight on the smooth running of the project and adherence to complete and correct reporting of all patients.

All data on patients age, complaint, diagnosis and prescription was recorded in the patient registers. The laboratory also kept a daily log sheet of all patients seen at the laboratory. The treatment outcome was finally recorded once the patient reached the endpoint in the study.

Retrospective parasite data for the facility from 2003 to 2008 was also reviewed in order to understand trends in parasite prevalence over the years.

### Data entry and analysis

The data was entered and analysed in Microsoft Office Excel 2007. Data was entered by data clerks with a clinical background to ensure that all information was well entered. The clinical officer and scientific officer provided supervision to the data entry clerk. The entries were verified and corrections made on a record by record basis. Frequencies were generated for the parameters under investigation. The analysis included generation of disease burden, prescription practices and patient treatment outcome.

The study was approved by the Ethics Committee of the Tropical Disease Research Centre. Patient data was recorded by study numbers. All information was kept by the project staff. However, the study was also obliged to provide accurate reports to the facility HMIS to ensure that no cases were missed when the facility was reporting on the indicators required.

## Results

### Malaria burden

The number of children under five who sought care from the facility between December 2007 and February 2008 was 1,383. These children came from the different regions of the catchment area of Chongwe rural health centre in Chongwe district. However, five children were excluded from analysis as there was no record of their reason for visiting the health facility, leaving a total analysable sample of 1,378. The average age for children visiting the health facility was 20.4 months (about one year and six months). The average temperature for all visits was 38.0°C.

Of the 1,378 children under five years, who were enrolled or visited the health facility, 0.7% (10/1378) were found positive for malaria and treated with artemether-lumefantrine (the first-line treatment for Zambia). 15 children who were negative for malaria were also treated with anti-malarials (13 received artemether-lumefantrine, while two received sulphadoxine-pyrimethamine). The two children who were treated with SP were three and seven months old, respectively. Thus, 1.8% children were treated with an anti-malarial (25/1378) regardless of their malaria status. The average temperature for patients with malaria was 39.0°C (range 37.5°C to 40.2°C) and the average age was 30 months (range 12 to 58 months).

A sample of 200 slides was taken for validation by two independent laboratory technicians. There was no disagreement with the result of the test for both positive and negative rapid diagnostic test result. The RDT used was the histidine-rich protein II (HRP-2) type, ICT Mal Pf. by ICT Diagnostics of South Africa.

Microscopy examination of the 10 positive slides did not show any mixed infections, only mono infections with *Plasmodium falciparum *were detected. Further, none of the patients who reported to the health facility had severe malaria and no deaths due to malaria were reported during the study period. Table 1 outlines the monthly visits and malaria parasite rates. More visits were reported in January due to high cases of skin infections and non-bloody diarrhoea in three areas of Chongwe (Dam area, Township and Libuko) in January alone.

**Table 1:** Facility visits for and malaria treatment

Month	Number of visits for Under-five	Number positive (Parasite prevalence)	Total anti-malarial treatments
December 2007	378	2 (0.53%) *95% CI [0.2–1.9]*	6 (1.6%) *95% CI [0.7–3.4]*
January 2008	638	7 (1.1%) *95% CI [0.5–2.2]*	13 (2.0%) *95% CI [1.2–3.4]*
February 2008	362	1 (0.3)% *95% CI [0.01–1.5]*	6 (1.7%) *95% CI [0.8–3.4]*
Total visits	1378	10 (0.7%) *95% CI [0.4–1.3]*	25 (1.8%) *95% CI [1.2–2.7]*

### Causes of outpatient visits

Acute respiratory infections (ARI) non-pneumonia (42.5%) and pneumonia (5.7%) were among the top five causes of morbidity at the facility. Malaria was not even among the top 10 causes of visitation at this facility (ranked number 15 (0.7%). Among the least causes of morbidity were diseases such as one case of tuberculosis (29 months) and one case of chicken pox was reported in the three months. Fever of unknown origin was found in 20 patients (1.5%), however, these patients were treated with paracetamol and no further complaints were received from parents or caretakers within the 14 days of follow up. Another 34 children (2.5%) were found not to have any illness and therefore did not need to visit the facility at all. However, they were treated with either paracetamol or some antibiotics.

### Referrals

A total of five children were referred for further management to the University Teaching Hospital. The referrals included one each for skin infection, severe malnutrition, fractured arm, conjunctivitis and the only case of tuberculosis. Three children were locally admitted at the health centre, two for pneumonia and one for severe diarrhoea.

### Treatment characteristics

Piriton was mostly prescribed than all the treatments as shown in table 2. Amoxyl was the most prescribed antibiotic (20%), followed by septrin (10.5%) and pen V (9.1%). Anti-malarials were prescribed in less than 2% of the patients, 23 patients were treated with Coartem^® ^(artemether-lumefantrine) and two with Fansidar^® ^(SP). All the patients treated with anti-malarials were not prescribed an antibiotic, but paracetamol as per standard treatment guidelines for patients with no concomitant illness. Other drugs included treatment for burns, sprain, worms, etc.

**Table 2:** Proportion of patients treated with a named drug

**Treatment**	**Number**	**Frequency (%)**
Piriton	396	28.7
Amoxyl	274	19.9
ORS	151	11.0
Septrin	145	10.5
Pen V	125	9.1
Panadol	114	8.3
Flagyl	48	3.5
Coartem	23	1.7
Hydrocortisone	22	1.6
Nystatin	17	1.2
Phenergan	13	0.9
Cephalexin	10	0.7
Erythromycin	9	0.7
Clotrimazole	8	0.6
Procaine penicillin	7	0.5
Other	16	1.2

**Total**	**1378**	

About 23.4% (323/1378) of all patients were prescribed with a single drug only. Among the 323 patients who received a single drug, 35% were treated with paracetamol alone, followed by Piriton^®^, Oral Rehydration Salts (ORS) and Amoxyl^® ^respectively as shown in Table 3. Five patients were treated with Coartem^® ^only (1.5%).

**Table 3:** Characteristics of single primary prescriptions

**Single prescriptions**	**Number**	**%**
Panadol	114	35.3
Prirton	63	19.5
ORS	40	12.4
Amoxyl	36	11.1
Pen V	23	7.1
TEO	14	4.3
Septrin	12	3.7
Phernergan	5	1.5
Coartem^®^	5	1.5
Cefalexin	4	1.2
Ciproflaxin	4	1.2
TSO	1	0.3
X-Pen	1	0.3
Griseofulvin	1	0.3

**Total**	**323**	

It was also observed that 77% of all the patients were prescribed at least two drugs. Paracetamol and a given treatment was a common combination accounting for 79% of the double prescriptions (prescriptions of two drugs). None of the patients were prescribed more than one antibiotic. However, it is possible that some patients may have been prescribed paracetamol in addition to the two antibiotics and this may not have been documented.

### Trends in parasite prevalence among under-five children with fever at OPD

The parasite prevalence among fever patients has been reducing from a high of 83% in 2004 to 0.7% in 2008 [22]. Further, population surveys have also shown that there has been a reduction in parasite prevalence among the under-five population from household surveys from 20% in 2006 to 11% in 2008 [18,19].

## Discussion

**Figure 1:**
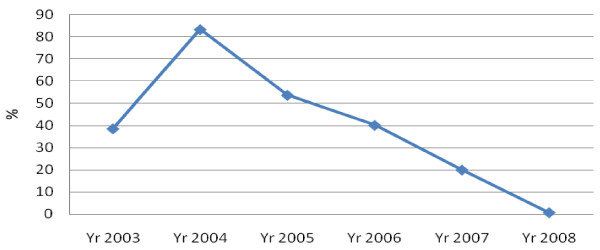
**Trends in parasite rates among fever patients at Chongwe health facility 2003–2008**.

The study has shown parasite rates lower than the national average. The health facility is situated in a district where an integrated package for malaria control has been implemented. The parasite prevalence at OPD was less than 1%, only 10 confirmed cases in three months during the rainy season. Earlier parasite survey results conducted at the same facility in January and February 2006 among under-five children, recorded a parasite prevalence of 28% at OPD, 7.9% among pregnant women and 7.0% among asymptomatic primary school children (aged 2–14 years) at Chongwe basic school [23]. Further, in 2005, out of the 3338 all OPD visits, malaria was confirmed in 35% of all patients with fever, while in 2006, malaria accounted for 24% of health facility visits at Chongwe rural health facility [24]. So it appears that the malaria disease burden at Chongwe rural health facility has been on a steady decline as shown in figure 1.

The availability of diagnostic facilities (either RDT or microscopy) created an opportunity to correctly identify malaria and non-malaria cases. This is an important step in improving not only malaria management, but overall patient management. The training which was given to the health workers before the study seemed to have assisted with using the diagnostic results to inform decision to prescribe an anti-malarial. Previous studies in Zambia showed that health workers still prescribed anti-malarials to more than 50% of negative cases [25].

Respiratory infections were diagnosed in more than 40% of the children under five years of age during the study period. It was not possible in this study to assess the quality of these diagnoses which were made for non-malaria fevers. However, the supervision reports for medical supervisors showed that patients were managed as per standard treatment guidelines. As malaria-related fever are reduced, the management of fever in under-five children is an area of further research to help identify gaps or document best practices where these may exist. This also has implications for home-management of fever using community health workers. The management of malaria and pneumonia studies are a necessary area for implementation research involving community health workers.

As seen from the prescription practices in this study, the use of antibiotics will increase to replace over prescription of anti-malarials at health facility level. Therefore, systematic antimicrobial resistance monitoring to these drugs should be developed. However, it was satisfying to note that paracetamol was prescribed in cases which were found not to need any medication as opposed to the use of antibiotics.

This study has also demonstrated that improved case management reduces severe malaria or severe patient outcomes. The health facility had effective first line treatment as per treatment policy in Zambia and diagnostic tools were readily available not only to the laboratory technician, but also to the nurses in the screening room. There was no severe malaria and no deaths reported among all the under-five children. Further, none of the referrals were malaria related. The community health worker was very useful in patient follow ups and feedback to the facility.

Some of the observation from this study is that the health system stands to potentially benefit from integrated malaria control through reduced malaria related revisits, reduced referrals, reduction in anti-malarial needs and in the long run reduction in health worker work load. However, the magnitude of these benefits need to be quantified by economic studies. Other studies in Africa have shown that rapidly scaling up coverage of effective treatment and vector control reduces malaria morbidity and mortality as shown in South Africa [26] and Zanzibar [27].

The study findings also seem to challenge the widely used indicator: "proportion of children with fever who received an anti-malarial". From this study, one could see that very few patients with fever were prescribed an anti-malarial because they really did not need to. So the indicator as it stands is of little use for programme managers and district personnel. However, it is important to measure among the "true malaria" cases, how many received an anti-malarial (or ACT). This will be important as more and more diagnostics are being scaled-up.

Implementing integrated malaria control implies investing in changing health worker prescription practices based on evidence based development of treatment guidelines.

The study's main limitation is the lack of assessment of the accuracy of non-malaria diagnoses. However, as can be seen from the study there were no adverse health outcomes.

## Conclusion

Implementing integrated malaria control has the potential to reduce malaria related visits at the health facility. However, respiratory infections are still prevalent. These findings have implications for the management of non-malaria fevers in children under the age of five years. Management of fever in under-five children needs updating based on the changes in epidemiology. As malaria will no longer be the suspect, potential mismanagement of non-malaria fevers is an important area of research. Improved health facility surveillance will be useful in redefining disease priorities in a given locality.

## Competing interests

The authors declare that they have no competing interests.

## Authors' contributions

PC designed the study protocol, supervised data collection, analysis and drafted the manuscript. BH was part of the protocol development, field supervision, data entry and provided input during manuscript development. VC trained field staff, supervised field work and participated in manuscript development. ECK s revised the protocol, supervised the field work and provided input in the manuscript development. CM supervised data collection, clinical management and participated in the manuscript development. SM participated in data collection, supervised data management and provided input on the manuscript.
